# Predictors of hypertension urgency in primary aldosteronism patients during the first 24 hours after surgery

**DOI:** 10.18632/oncotarget.21632

**Published:** 2017-10-07

**Authors:** Juping Zhao, Jun Dai, Wenlong Zhou, Haofei Wang, Wenbin Rui, Wei He, Zhe Zhu, Yu Zhu, Danfeng Xu, Fukang Sun

**Affiliations:** ^1^ Shanghai JiaoTong University School of Medicine, Ruijin Hospital, Department of Urology, Shanghai, 200025 China; ^2^ Department of Medicine, Division of Regenerative Medicine, University of California, San Diego, School of Medicine, La Jolla, CA, 92037 USA; ^3^ Department of Stem Cell Biology and Regenerative Medicine, Lerner Research Institute, Cleveland Clinic, Cleveland, OH, 44195 USA

**Keywords:** aldosterone-producing adenoma, predictor, hypertension urgency, laparoscopic adrenalectomy

## Abstract

Study about blood pressure variation in the first 24 hours post-operation is limited in patients with adrenal aldosterone-producing adenoma. We aim to evaluate the potential predictors for postoperative hypertension urgency during the first 24 hours after laparoscopic adrenalectomy in patients with aldosterone-producing adenoma. Clinical data of 177 patients with aldosterone-producing adenoma were retrospectively collected from January 2009 to December 2015 and the potential factors that may influence postoperative blood pressure during the first 24 hours after surgery were analyzed. The factors included gender, age, body mass index, preoperative maximum systolic blood pressure, number of antihypertensive medicines, preoperative spironolactone treatment, duration of hypertension, surgical method and approach, adenoma diameter, preoperative proteinuria, estimated glomerular filtration rate, serum potassium and serum aldosterone. Univariate and multivariate regression analyses were used to evaluate the relationship between the above variables and postoperative hypertension urgency. We found that the proportion of patients with a higher systolic blood pressure ≥ 160 mmHg and ≥ 180 mmHg were significantly increased post-operation (both *p* < 0.001). In multivariate analysis, the maximum systolic blood pressure was an independent predictor of postoperative hypertension urgency, and the cut-off point was 157 mmHg with the sensitivity of 66% and specificity of 82%. Multivariable analysis also showed that preoperative maximum systolic blood pressure and number of antihypertensive medicines were independent risk factors for higher postoperative systolic blood pressure. This study was derived from a high volume adrenal tumor center, and these data may provide a potential tool to guide preoperative counseling.

## INTRODUCTION

Among the general hypertensive population, primary hyperaldosteronism (PHA) is widely recognized as the most common cause of secondary hypertension [1]. About 3–10% of hypertension patients are diagnosed with PHA [2, 3], with a higher rate of cerebrovascular complications [4]. Therefore, PHA is a recent research hotspot. PHA is caused by the hypersecretion of aldosterone hormone due to adrenocortical lesions, and is associated with clinical manifestations of hypertension and hypokalemia. There are two subtypes of PHA, aldosterone-producing adenoma (APA) and idiopathic hyperaldosteronism (or bilateral adrenal hyperplasia). APA accounted for about 60% of PHA [5]. Hypokalemia can be successfully cured in most APA patients by surgery, which includes partial adrenalectomy and unilateral total adrenalectomy [6, 7]. However, the recovery of blood pressure(BP) is still facing challenge in clinical setting. There are significant variations in BP during the first 24 hours after surgery, which can cause acute severe cerebrovascular complications, such as stroke, cardiac infarction, etc.

Numerous studies have identified factors associated with long-term outcomes after adrenalectomy for PHA [8–10]. However, knowledge of BP variations in the first 24 hours post-operation is limited. In this study, we evaluated the potential predictors for postoperative hypertension urgency during the first 24 hours after laparoscopic adrenalectomy (LA) in patients with APA.

## RESULTS

### Clinical demographics

A total of 177 patients with APA were enrolled in this study. The demographic characteristics of the patients are shown in Table 1. The median age was 46 (IQR = 38–53) years, and 44.6% of the patients were male. The median duration of preoperative hypertension was 5.5 years (IQR = 2–10 years). Preoperative evaluation indicated that the patients were on a median of two antihypertensive medicines, with a median maximum systolic blood pressure(SBPmax) of 140 mmHg (IQR = 130–158 mmHg).

**Table 1:** Characteristics of 177 patients with adrenal aldosterone-producing adenoma

Variable	Value
No. of males (%)	79 (44.6%)
Age of operation (years) (Median, IQR)	46 (38–53)
BMI (kg/m^2^) (Median, IQR)	23.4 (21.5–25.5)
Duration of hypertension (years) (Median, IQR)	5.5 (2.0–10.0)
Preoperative SBPmax (mmHg) (Median, IQR)	140 (130–158)
Number of preoperative antihypertensive medicine(Median, IQR)	2 (2–3)
Surgery type	
Partial adrenalectomy	109 (61.6%)
Total unilateral adrenalectomy	68 (38.4%)
Surgery approach	
Transperitoneal	70 (39.5%)
Retroperitoneal	107 (60.5%)
Operating time (min) (Median, IQR)	105 (85–130)
Adenoma diameter (cm) (Median, IQR)	1.5 (1.2–2.0)
Preoperative eGFR (mL/min/1.73m^2^) (Median, IQR)	102 (84–118)
Preoperative serum aldosterone (ng/dL) (Median, IQR)	466 (306–700)
Preoperative serum potassium (mmol/L) (Median, IQR)	3.9 (3.6–4.1)
Postoperative blood pressure (mmHg) (Median, IQR)	160 (150–170)

**Figure 1:**
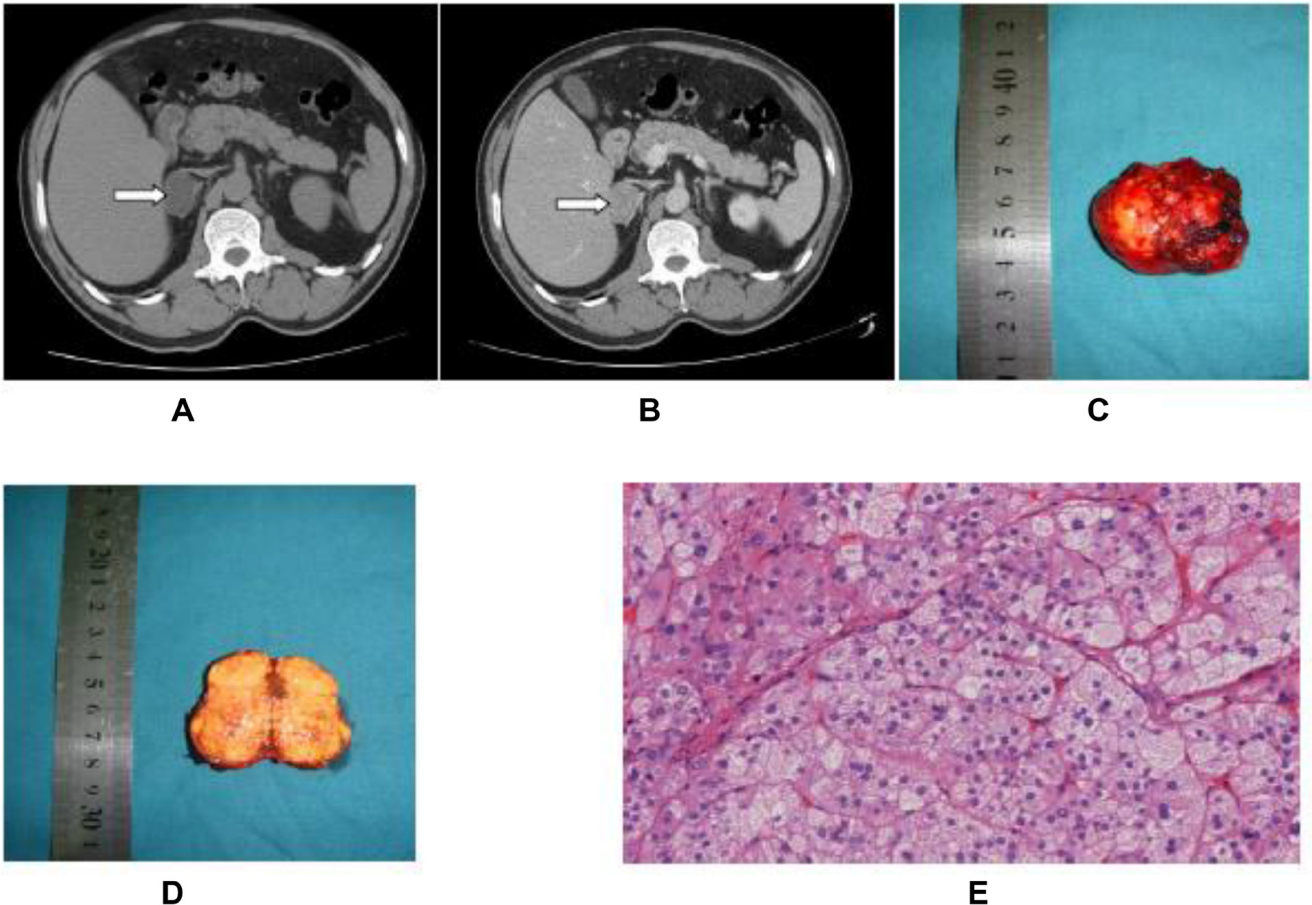
Primary hyperaldosteronism in a 59-year-old male patient (**A** and **B**) CT image (plain and contrast) of primary adrenal aldosterone-producing adenoma. (**C** and **D**) Specimen picture of aldosterone-producing adenoma. (**E**) H & E staining of the aldosterone-producing adenoma (10 × 10).

Laparoscopic partial or total adrenalectomy was successfully performed in all patients, 61.6% and 38.4%, respectively, without conversions to open surgery. Surgery was conducted by the transperitoneal approach in 39.5% of cases and retroperitoneal approach in 60.5% of cases. All 177 cases were pathologically confirmed to be benign adrenal cortical adenoma. There were no deaths or severe complications during hospitalization. The CT, specimen and pathology images of a typical APA case are shown in Figure 1.

The median concentration of serum aldosterone at diagnosis was 466 pg/mL (IQR = 306–700). The normal value of serum aldosterone at our center was 38–313 pg/ml in standing position. Thirty-one patients (17.5%) received intravenous antihypertensive medication in the immediate postoperative period because of persistent excessively high BP.

### Trend of blood pressure variations

The systolic blood pressure(SBP) increased by a median of 20 mmHg (IQR = 5–28 mmHg) post-operation, and the median percentage was 13.4% (IQR = 3.3–20.4%). There were 91 patients (51.4%) with BP ≥ 160 mmHg post-operation as compared to 40 patients (22.6%) pre-operation, *p* < 0.01. Hypertension urgency was seen in 29 (16.4%) patients after surgery, with a significant increase from 11 cases (6.2%) pre-operation.

### Factors associated with hypertension urgency in the first 24 hours post-operation

When postoperative hypertension urgency was considered as a categorical variable for univariate analysis, preoperative SBPmax (*p* = 0.001), duration of preoperative hypertension (< 5 years) (*p* = 0.001), surgical method (*p* = 0.043), and preoperative serum potassium (< 3.5 mmol/L) (*p* = 0.010) were associated with postoperative hypertensive urgency (Table 2).

**Table 2:** Univariate analysis of risk factors for postoperative hypertension urgency

Variable	NPHU group	PHU group	*P*
Male	67/148 (45.3%)	12/29 (41.4%)	0.702
Age of operation (years) (Median, IQR)	46 (38–52)	50 (39–56)	0.388
BMI (kg/m^2^) (Median, IQR)	23.5 (21.6–25.4)	22.7 (21.5–26.5)	0.625
Preoperative SBPmax (mmHg) (Median, IQR)	140 (130–150)	160 (150–173)	0.001^**^
Number of preoperative antihypertensive medicine (Median, IQR)	2 (2–3)	2 (2–3)	0.393
Preoperative spirolactone treatment	126/148 (85.1%)	23/29 (79.3%)	0.435
Duration of hypertension < 5 years	72/148 (48.6%)	4/29 (13.8%)	0.001^**^
Surgical method, partial	96/148 (64.9%)	13/29 (44.8%)	0.043^*^
Surgical approach, transperitoneal	59/148 (39.9%)	11/29 (37.9%)	0.847
Adenoma diameter (cm) (Median, IQR)	1.5 (1.2–2.0)	1.5 (1.2–2.0)	0.856
Preoperative proteinuria	5/148 (3.4%)	2/29 (6.9%)	0.377
Preoperative eGFR (ml/min/1.73 m^2^) (Median, IQR)	101 (83–115)	111 (88–124)	0.251
Preoperative serum potassium (mmol/L) (< 3.5 mmol/L)	17/148 (11.5%)	9/29 (31.0%)	0.010^*^
Preoperative serum aldosterone (pg/ml)	467 (294–686)	451 (342–766)	0.215

NPHU: non-postoperative hypertensive urgency; PHU: postoperative hypertensive urgency. ^*^p < 0.05, ^**^p < 0.01

**Figure 2:**
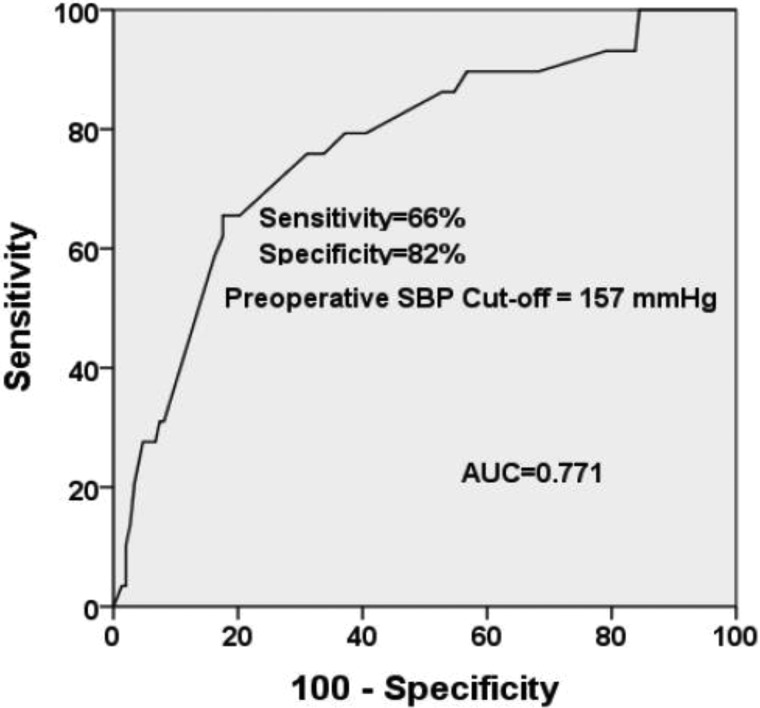
ROC curve indicated that preoperative SBPmax can accurately predict the occurrence of postoperative hypertensive urgency The cut-off point was 157 mmHg, with the sensitivity of 66% and specificity of 82%.

In multivariate analysis, preoperative SBPmax (*p* = 0.001) was the only independent prognosticator (HR: 0.180, 95% confidence interval 0.079–0.281). When the ROC curve of preoperative SBPmax was generated, the area under the curve was 0.771, consistent with good fit to the postoperative hypertension urgency data (Figure 2). The preoperative SBPmax of 157 mmHg was the cut-off point, with 65.5% sensitivity and 82.4% specificity for postoperative hypertension urgency.

On univariate analysis, preoperative SBPmax (*p* = 0.000), number of antihypertensive medications (*p* = 0.032), duration of preoperative hypertension (< 5 years) (*p* = 0.007), and serum potassium (< 3.5 mmol/L) (*p* = 0.015) were associated with postoperative higher BP as a continuous variable (Table 3). Multivariate logistic regression analysis was performed on factors identified in univariate analysis, and showed that preoperative SBPmax (*p* = 0.045) and number of antihypertensive medications (*p* = 0.023) were independent prognosticators.

**Table 3:** Univariate analysis of risk factors for higher postoperative SBP

Variable	Value	*P*
Male	44.6%	0.161
Age of operation (years) (Median, IQR)	46 (38–53)	0.081
BMI (kg/m^2^) (Median, IQR)	23.4 (21.5–25.5)	0.351
Preoperative SBPmax (mmHg) (Median, IQR)	140 (130–158)	0.000^**^
Number of preoperative antihypertensive medicine (Median, IQR)	2 (2–3)	0.032^*^
Preoperative spirolactone treatment	84.2%	0.224
Duration of hypertension (< 5 years)	42.9%	0.007^**^
Surgical method (partial)	61.6%	0.140
Surgical approach (transperitoneal)	39.5%	0.445
Adenoma diameter (cm) (Median, IQR)	1.5 (1.2–2.0)	0.421
Preoperative proteinuria	4%	0.294
eGFR (ml/min/1.73 m^2^) (Median, IQR)	102 (84–118)	0.883
Preoperative serum potassium (< 3.5 mmol/L)	14.7%	0.015^*^
Preoperavite serum aldosterone (pg/ml)	466 (306–700)	0.275

^*^p < 0.05, ^**^p < 0.01.

## DISCUSSION

Hypertension can cause numerous comorbidities, and is the leading preventable risk factor for death in recent decades [11, 12]. PHA is widely recognized as one of the most common causes of secondary hypertension, and persistent hypertension causes targeted organ dysfunction [4]. Monticone and colleagues reported that 5.9% patients were diagnosed with PHA among 1,672 primary hypertension patients [1]. APA is a subtype of PHA that can be cured by adrenalectomy [6, 7]. Most studies focus on the predictive factors of long-term outcomes after adrenalectomy [13, 14]. Older age, longer duration of hypertension, increased serum creatinine, preoperative use of > 2 antihypertensive medicines, and positive family history of hypertension were reported as predictors of persistent hypertension after adrenalectomy in PHA population [13, 14]. Clinically, BP varies significantly during the first 24 hours post-operation. To our knowledge, in the setting of APA, BP variations during the first 24 hours post-operation has not been well studied. Therefore, we conducted this study to evaluate the potential predictors for postoperative hypertension urgency within the first 24 hours after LA in patients with APA.

High BP induced by hyperaldosteronism can be exacerbated in the immediate postoperative period by factors such as pain, hypercapnia, hypoxia, intravascular volume overload and hyperthermia [15, 16]. In addition, oral antihypertensives are contraindicated during the first 24 hours post-operation due to temporary impairment of digestive function. Therefore, postoperative hypertension urgency was frequently observed after laparoscopic adrenalectomy in patients with APA. Though the progress of anesthesia was fixed and similar at our center and adrenalectomy was performed by experienced surgeons, the median percentage of SBP ≥ 160 mmHg increased significantly from 22.6% pre-operation to 51.4% post-operation. The median percentage of SBP ≥ 180 mmHg increased from 6.2% to 16.4%, *p* < 0.01. In the 29 patients with SBP ≥ 180 mmHg post-operation, intravenous antihypertensive drugs were immediately administered when oral therapy could not be administered. Nicardipine Hydrochloride injection (0.5–6 ug/kg/min) or Urapidil Hydrochloride injection (2 mg/min) were the primary therapeutic options at our institution. Drip speed adjustment was made according to the real-time SBP. Timely intervention led to safe and efficient outcome, without cerebrovascular complications.

There was a trend of increase in BP after surgery due to several factors. Wachtel and colleagues evaluated several protective factors of persistent postoperative hypertension, including female gender, BMI (< 25 kg/m^2^), hypertension (< 5 years duration), serum creatinine (< 0.8 mg/dL), and < 2 preoperative antihypertensive medicine [8]. In this study, we evaluated the potential factors that could increase the postoperative SBP within 24 hours in patients with APA. In the multivariable analysis, using postoperative SBPmax as a continuous variable, preoperative SBPmax and number of antihypertensive medicines were the independent prognosticators, HR 6.295 and 3.387, respectively (both *p* < 0.05). The number of antihypertensive medicines was a meaningful parameter to predict the immediate and long-term BP variations. Considering SBPmax ≥ 180 mmHg as a categorical variable (whether hypertension urgency or not), only preoperative SBPmax was an independent prognosticator, *p* = 0.001.

Actually, there are many additional factors which could affect BP variation after surgery, such as anesthesia. In our study, the BP influence of anesthesia exists objectively, though the progress of anesthesia was fixed and similar. This is one of the limitation

SBP is considered as an important parameter in the hypertensive population with prevalent cerebrovascular diseases [17]. Recent studies provided clinical evidence for a target SBP in elderly patients that was significantly associated with high risk of cardiovascular events [18]. So we propose preoperative SBPmax as a potential predictor of hypertension urgency in patients with APA within the first 24 hours post-operation. Additionally, we generated the ROC curve for further analysis and found that preoperative SBPmax of 157 mmHg was the cut-off point, whose sensitivity was 66% and specificity was 82%.

In our study, gender, BMI, duration of hypertension and preoperative serum creatinine were not significantly associated with postoperative hypertension urgency in multivariate analysis. The morbidity of APA was similar between men and women in our cohort, and there was no significant variation in BMI (median 23.4, IQR:21.5–25.5) due to Asian ethnicity. Interestingly, we found that duration of hypertension was not a predictor for postoperative immediate SBP, which was inconsistent with previous reports on long-term hypertension [13, 14]. Due to limited sample size in our study, further research is needed in this field, and post-operative BP should be followed in the longer term in these patients.

In conclusion, our data derived from the high volume adrenal tumor center and the results are relatively representative in population. The preoperative SBPmax was independently associated with hypertension urgency within the first 24 hours after laparoscopic adrenalectomy. These data may provide a potential tool to guide preoperative counseling. Control of preoperative SBP could decrease the occurrence of hypertension urgency within the first 24 hours post-operation.

## MATERIALS AND METHODS

### Study population

After approval by the Ethics Committee of Ruijin Hospital, the adrenal surgery registry database was used to identify patients who were managed with LA from January 2009 to December 2015. During this time, clinical data of 177 patients with APA were retrospectively collected at our adrenal disease center. Written informed consent was obtained from all enrolled patients. Before surgery, the patients underwent an examination in the department of Hypertension or Endocrinology. The diagnosis of PHA was based on endocrinological evaluation, preoperative imaging studies (CT or MRI) and postoperative pathology. Before endocrinological evaluation, mineralocorticoid receptor antagonists were washed-out for at least six weeks. Other antihypertensive drugs were replaced with verapamil (Isoptin) sustained-release tablets for at least two weeks. A positive diagnosis for PHA was based on elevated serum aldosterone level with increased aldosterone-to-renin ratio, and was confirmed by the absence of aldosterone suppression after a saline-loading test. All serum aldosterone concentrations were measured in a standing position, with normal value of 38–313 pg/mL at our center. Adrenal venous sampling (AVS) was conducted to distinguish between unilateral and bilateral adrenal lesions. Patients who refused to undergo AVS were excluded from this study. Each paraffin section and hematoxylin-eosin (HE) stained section was independently evaluated by two pathologists using standard criteria based on the WHO classification. The third independent pathologist adjudicated the section in case of different opinions.

### Surgery

Laparoscopic total or partial adrenalectomy was performed via a standard transperitoneal approach in 70 patients and a retroperitoneal approach in 107 patients. The decision to perform a partial or total unilateral adrenalectomy was intraoperatively made based on the position of the adenoma in the adrenal gland and whether concomitant multiple microadenomas and nodules were presented [19, 20]. Laparoscopic partial adrenalectomy was preferred when the adenoma was eccentric, without multiple microadenomas and nodules. In laparoscopic partial adrenalectomy, the adrenal central vein was preserved if possible to ensure sufficient blood drainage to the remnant gland. Laparoscopic total unilateral adrenalectomy was imperative when the adenoma was near the core of the gland or when multiple microadenomas and nodules were detected. In this study, the operation time covered the period from anesthesia start to anesthesia recovery.

### Potential risk factors

The potential risk factors included gender, age, body mass index (BMI), preoperative maximum systolic blood pressure (SBPmax), number of antihypertensive medicines, preoperative spironolactone treatment, duration of hypertension, surgical method and approach, adenoma diameter, preoperative proteinuria, estimated glomerular filtration rate (eGFR), serum potassium and serum aldosterone. BP was measured by the adjusted electronic sphygmomanometer routinely in our ward at least three times a day before surgery and then noted in the medical records. The preoperative highest SBP value was considered as a potential risk factor. Postoperative SBP was obtained via electrocardiogram monitors (Boeblingen GmbH, Philips Inc., Germany) as soon as the patients were transferred back to the wards during the first 24 hours post-operation. Semiautomatic noninvasive measurements of BP were performed every half an hour in the supine position. In this study, hypertension urgency was defined as SBP ≥ 180 mmHg [21], which typically results in headache, dizziness, vomiting and convulsions. Postoperative hypertension was considered if SBP ≥ 160 mmHg. We checked the patient's medical history to determine the number of antihypertensive medicines, whether or not preoperative spironolactone treatment was administered, and duration of hypertension. Proteinuria was defined as a urine protein concentration > 30 mg/dL, and eGFR was calculated using the following equation developed and validated based on Chinese population: eGFR (mL/min/1.73 m^2^) = 175× (serum creatinine)–1.234 × (age)–0.197(× 0.75 in case of women) [22]. Preoperative serum potassium and aldosterone levels were detected by routine lab tests at our institute.

### Statistical analysis

Group comparisons were made using the Students’ *t*-test, Chi-square test, or Wilcoxon rank-sum test, as appropriate. Univariate analysis of risk factors for postoperative hypertension urgency and higher SBP was performed to detect the significant prognosticator. Then, multivariate analysis was used to evaluate the independent prognosticator. To dichotomize continuous variables, receiver operating characteristic (ROC) curves of independent prognosticators were generated, and values with maximal sensitivity were identified. The differences were considered significant if *p* < 0.05. Data were analyzed by using SPSS version 20.0 (SPSS Inc., Chicago, IL, USA).
